# Efficient Deterministic Finite Automata Minimization Based on Backward Depth Information

**DOI:** 10.1371/journal.pone.0165864

**Published:** 2016-11-02

**Authors:** Desheng Liu, Zhiping Huang, Yimeng Zhang, Xiaojun Guo, Shaojing Su

**Affiliations:** College of Mechatronics and Automation, National University of Defense Technology, ChangSha 410073, China; Shanxi University, CHINA

## Abstract

Obtaining a minimal automaton is a fundamental issue in the theory and practical implementation of deterministic finite automatons (DFAs). A minimization algorithm is presented in this paper that consists of two main phases. In the first phase, the backward depth information is built, and the state set of the DFA is partitioned into many blocks. In the second phase, the state set is refined using a hash table. The minimization algorithm has a lower time complexity *O*(*n*) than a naive comparison of transitions *O*(*n*^2^). Few states need to be refined by the hash table, because most states have been partitioned by the backward depth information in the coarse partition. This method achieves greater generality than previous methods because building the backward depth information is independent of the topological complexity of the DFA. The proposed algorithm can be applied not only to the minimization of acyclic automata or simple cyclic automata, but also to automata with high topological complexity. Overall, the proposal has three advantages: lower time complexity, greater generality, and scalability. A comparison to Hopcroft’s algorithm demonstrates experimentally that the algorithm runs faster than traditional algorithms.

## Introduction

Finite automata, regular grammar, and regular expressions are three dissimilar representations for regular languages. Regular grammar and regular expressions generate regular languages, and finite automata is a computation model of speech recognition for regular languages [1]. Finite automata is widely used in areas such as text processing [2], compilation [3], pattern matching [4, 5], network intrusion detection and protection [6, 7], image analysis and spatial dynamics [8, 9]. Finite automata mainly have two alternative implementations: nondeterministic finite automatons (NFAs) and deterministic finite automatons (DFAs). DFAs are used widely because they have predictable and acceptable memory bandwidth requirements. Regular expressions are usually used to describe engineering requirements in practice, and they can be converted into a DFA by a sequence of operations [10, 11]. To save memory space, the minimization of a DFA is indispensable in practical applications. In this paper, we focus on the minimization problem without considering the conversion from regular expressions or regular grammar to DFA.

It has been proven theoretically that a DFA has a unique minimal formalization, but only up to isomorphism [3]. This means that the minimal DFA is unique, and has the least number of states needed to recognize a language represented by regular expressions or regular grammar. The minimization of a DFA is beneficial not only for practical applications, but also mathematical problems in theory. Many algorithms can be used to minimize a DFA, and these can be classified into four categories.

The first category uses a series of refinements of a partition on the state set. A typical method is mentioned in [12], and has time complexity *O*(*n*^2^). Hopcroft’s and Moore’s algorithms also fall into this category. At present, Hopcroft’s algorithm is the most efficient known algorithm and has a worst-case time of *O*(*n* log *n*) [13]. Several authors [14, 15] have proven the tightness of the upper bound of this complexity for different families of automata. Hopcroft’s algorithm has recently been extended to handle incomplete DFAs [16, 17], and has now been re-described many times to make it easier to understand [18, 19]. Moore’s algorithm is much simpler than Hopcroft’s algorithm. However, it leads to a quadratic worst-case time complexity [20, 21].

The second type of minimization algorithm uses a sequence of merging states. The most famous algorithm of this type is Revuz’s algorithm, which has a linear time complexity. The disadvantage of Revuz’s algorithm is that it is constrained to acyclic automata [22]. Almeida and Zeitoun extended it to automata whose nontrivial strongly connected components are cycles [23, 24].

In the third category, Brzozowski’s algorithm reverses and trims the automaton twice to obtain the minimal DFA [25]. Recently, studies on automaton minimization algorithms have mainly focused on incremental construction and dynamic DFA minimization, which is the fourth classification. Daciuk proposed an incremental algorithm for minimizing finite state automata that allows the minimization to be merged with the construction in a single step by adding new strings one by one and minimizing the resulting automaton on-the-fly [26]. Another incremental determinization algorithm for general finite automata called ISC is presented in [27]. Finally, the dynamic minimization solution proposed by Carrasco and Forcada keeps an automaton minimal when insertions or deletions are performed [28].

The previously proposed approaches have two main drawbacks. On the one hand, previous methods cannot be used on complicated automata; they are only suitable for acyclic automata, local automata, or other special automata. Automata that have distinguishable accepted states are generally used in many applications, such as network intrusion detection and image analysis. However, only Hopcroft’s algorithm can minimize such DFAs. On the other hand, many algorithms are too complicated, slow, and difficult to implement. Along with the increases in size and topological complexity of DFAs in practice, the time consumed by minimization becomes increasingly important. In this paper, an efficient minimization algorithm is presented that can be used on various automata and is efficient in practice. The main contributions of this paper are as follows.

1. A minimization algorithm based on backward depth information is proposed. We define backward depth and prove the correctness of a partition based on this information.
2. Refinements based on a hash algorithm are introduced to obtain the final minimal DFA. The efficiency of this algorithm is explained and proven experimentally.
3. The proposed algorithm can obtain greater generality and time efficiency. Specifically, it can be efficiently implemented in scenarios where the automata has a more complicated topology complexity.

The remainder of this paper is organized as follows: firstly, some definitions and propositions related to the minimal DFA and proposed algorithm are stated. Then, we present the detailed steps for obtaining a minimal DFA: building backward depth information, hash table refinement with hash collision checking. In addition, the experiments and results for evaluating the efficiency of the algorithm are presented. Finally, we concludes the paper with a discussion on practical time efficiency, application range, and possible extensions.

## Preliminaries

In order to illustrate the proposed minimization algorithm more clearly, some preliminaries that relate to the minimization of DFA are presented. First, some related concepts are defined, such as DFA, minimal DFA, and partition [12, 29]. Next, a series of propositions that are used in the algorithm are introduced, and the proofs can be found in S1 File.

**DFA.** A DFA can be defined as a five-tuple *D* = < *Q*, Σ, *δ*, *q*_0_, *F* >, where

- *Q* is a finite set of DFA states.
- Σ is a finite set of input symbols.
- *δ*: *Q* × Σ → *Q* is a transition function.
- *q*_0_ ∈ *Q* is the initial state.
- *F* ⊂ *Q* is the accepted state set.

**Fig 1:**
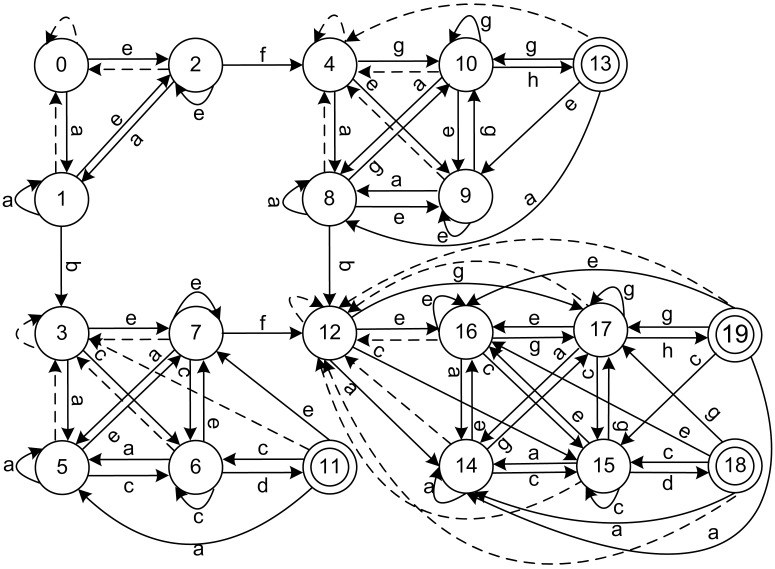
Original DFA state transition digraph for regular expressions (ab.*cd, ef.*gh). The numerical circle represents states in DFA, and the state with double circles represents it is an accepted state. The direct edges with a symbol represents transitions in DFA, and the dashed direct edges represents transitions transfer to a common state with symbols that have not been shown.

Given word *w* = *a*_1_ *a*_2_ ⋯ *a*_*n*_(*a*_1_, *a*_2_, ⋯, *a*_*n*_ ∈ Σ) as the input, the current state will be transformed into the sequence according to transition function *δ*. This transition function can be extended into a function $\hat{\delta }:Q\times \Sigma^{*}\to Q$, where $\hat{\delta }\left(p,aw\right)=\hat{\delta }\left(\delta \left(p,a\right),w\right)$, Σ* represents the set of words that composed by input symbols. If $\hat{\delta }\left(q_{0},w\right)\in F$, then word *w* is recognized. A DFA can be expressed in other formals including a state transition table or state transition digraph. The state transition digraph and state transition table for regular expressions *RE* = (*ab*. * *cd*, *ef*. * *gh*) are given in Fig 1 and Table 1, respectively. The dot-star notation “.*” in the regular expressions represents any number of repetitions of any character. Because there are too many edges in the graph for the automata, unlabeled dashed edges represent the transitions that have the same next state for different symbols that have not been shown. In Table 1, where *S* represents the states in the automata, the accepted states are bold, Σ denotes the symbol set, and the transitions are listed.

**Table 1:** State transition table for regular expressions (ab.*cd, ef.*gh).

Σ	a	b	c	d	e	f	g	h
S								
0	1	0	0	0	2	0	0	0
1	1	3	0	0	2	0	0	0
2	1	0	0	0	2	4	0	0
3	5	3	6	3	7	3	3	3
4	8	4	4	4	9	4	10	4
5	5	3	6	3	7	3	3	3
6	5	3	6	11	7	3	3	3
7	5	3	6	3	7	12	3	3
8	8	12	4	4	9	4	10	4
9	8	4	4	4	9	4	10	4
10	8	4	4	4	9	4	10	13
**11**	5	3	6	3	7	3	3	3
12	14	12	15	12	16	12	17	12
**13**	8	4	4	4	9	4	10	4
14	14	12	15	12	16	12	17	12
15	14	12	15	18	16	12	17	12
16	14	12	15	12	16	12	17	12
17	14	12	15	12	16	12	17	19
**18**	14	12	15	12	16	12	17	12
**19**	14	12	15	12	16	12	17	12

The function of DFA *D* is to recognize regular language *L*, and this can be formally expressed as $L\left(D\right)=\{w\in \Sigma^{*}|\hat{\delta }\left(q_{0},w\right)\in F\}$. However, there are many DFAs that have different numbers of states for a regular expression set. If two DFAs *D* and *D*′ recognize the same regular expression set, then *D* and *D*′ are equivalent, which is denoted as *L*(*D*) ≡ *L*(*D*′). In the sense of isomorphism, there exists a minimal DFA that has the fewest number of states. A minimal DFA is defined as follows.

**Minimal DFA.** If DFA *M* is minimal, then there is no other DFA *M*′ that has fewer states and is equivalent to DFA *M*.

The minimal DFA for any regular language is unique up to isomorphism, and this has been proved [3]. The transformation from a DFA to an equivalent minimal DFA is called the minimization. In a normal DFA, the essential cause of redundant states is that it has equivalent states. We present the definition of equivalent states as follows.

**Equivalent States.** Suppose that *p* ∈ *Q*, *q* ∈ *Q*, and *w* ∈ Σ* in DFA *D* = < *Q*, Σ, *δ*, *q*_0_, *F* > maintain $\hat{\delta }\left(p,w\right)=s_{i}$ and $\hat{\delta }\left(q,w\right)=s_{j}$. If states *s*_*i*_ and *s*_*j*_ are either both accepted states or non-accepted states, and *s*_*i*_ and *s*_*j*_ are equivalent when they are accepted states, then states *p* and *q* are said to be equivalent, which denoted as *p* ≡ *q*; otherwise, *p* and *p* are distinguishable(*p ≢ q*).

According to the above, the minimization of a DFA can be treated as a problem of determining whether any two states in the state set of the DFA are distinguishable or equivalent. The most straightforward approach to this problem is to examine the consistency of strings that reach accepted states from any two states, that is, verifying *L*_*p*_(*w*) ≡ *L*_*q*_(*w*) directly, where $L_{p}\left(w\right)=\left\{s|\hat{\delta }\left(p,w\right)\right\}$. However, the cost of doing so is great. For a cyclic automaton, it is impossible to enumerate all strings that can go from a certain state to an accepted state. Fortunately, the equivalence of any two states can be determined by the relationship between states or transitions. In this paper, the idea of a partition is adopted to obtain the minimal DFA. All states in the DFA state set are considered as a whole, and the nonequivalent states are found step by step. The minimal DFA is obtained when no equivalent states exist. To explain the algorithm more clearly, we define a partition as follows.

**Partition.** A partition of set *E* is a family *P* of nonempty, pairwise, disjoint subsets of *E* such that $E\;=\;{⋃}_{P_{k}\in P}\;P_{k}$. |*P*| represents the number of subsets in the partition *P*. If |*P*| is greater, the partition is more refined.

To partition the state set efficiently, the brute force method is discarded, and a series of propositions for distinguishing states are introduced.

**Proposition 1.** For states *p* ∈ *Q*, *q* ∈ *Q*, if there exists a symbol *a* that maintains *δ*(*p*, *a*) = *s* and *δ*(*q*, *a*) = *t* and furthermore, *s* and *t* are distinguishable, then *p* and *q* are distinguishable.

Proposition 1 proves that the partition can be obtained according to the transitions in the DFA; however, it is an inefficient way to obtain the minimal DFA directly. To improve the efficiency of the minimization, the backward depth is defined and its related propositions are introduced.

**Backward Depth.** The backward depth from state *p* to accepted state *t* is defined by the length of the shortest path from *p* to *t*, and can be formally denoted as $BD\left(p,t\right)=\min\{|w||\hat{\delta }\left(p,w\right)=t,t\in F\}$.

In other words, the backward depth is the short length of words consumed in the process of state p transfer to accepted state t. The backward depth information for the DFA can be constructed using the reversal DFA and algorithm proposed in the next section. The reversal DFA is defined as follows.

**Reversal DFA.** Reversal DFA *R* of DFA *D* is also represented using a five-tuple *R* = < *Q*, Σ, *δ*^−^, *q*_0_, *F* >. The unique difference between *R* and *D* is the reversal transition function *δ*^−^: *Q* × Σ → Q*, where *δ*^−^(*p*, *a*) = {*q*|*δ*(*q*, *a*) = *p*}, and Q* is the power set of *Q*.

The backward depth information can be used to coarsely partition the states according to the following proposition.

**Proposition 2.** If the backward depths of two states *p* and *q* for any accepted state *t* are different, *p* and *q* must be distinguishable. Formally, if *BD*(*p*, *t*) ≠ *BD*(*q*, *t*), then *p* ≢ *q*.

It is possible that the DFA has many accepted states, so we can obtain the backward depth information of one state to many different accepted states. The state set can be partitioned according to the backward depth information for each accepted state; thus, many partitions are generated. We can refine a partition using the backward depth information according the following proposition.

**Proposition 3.** Given two partitions *U* and *V* of set *E*, we say that *W* = *U* ∩ *V* refines *U* and *V*. This means if *U*_*i*_ and *V*_*j*_ are elements of *U* and *V*, respectively, then these two elements will be divided into three elements in *W*, i.e., *W*_*k*_ = *U*_*i*_ ∩ *V*_*j*_, *W*_*m*_ = *U*_*i*_\*W*_*k*_, and *W*_*n*_ = *V*_*j*_\*W*_*k*_.

**Fig 2:**
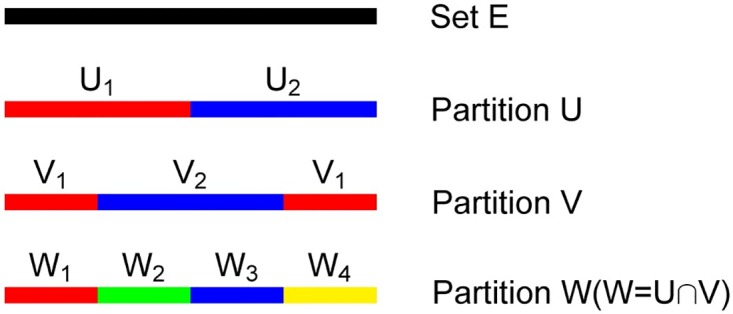
Example of obtaining a refined partition from coarse partitions. *U*,*V* and *W* represent different partitions for the set *E*. The bar with different colors represents a subset obtained by the corresponding partition, and the *W* partition is generated by the intersection of *U* and *V*.

To explain it clearly, an example of proposition 3 is given in Fig 2. Different colors represent different elements in the corresponding partitions. Partition *U* includes two elements represented by red and blue segments, and *V* also has two parts, colored red and blue. The result *W* is divided into four segments colored red, blue, green, and yellow.

Although a comparatively refined partition of a state set can be obtained using backward depth information, it may not be the final minimal DFA. To obtain the final minimal DFA, refinement using a hash table is presented in the next section.

## Minimal DFA Construction Algorithm

The minimization algorithm proposed in this paper solves the task in two main phases. In the first phase, the backward depth information for every accepted state is generated for each state, and the state set is grouped into many blocks. In the second phase, states in the same block are refined according to the hash table going from the deepest to the shallowest state.

### Coarse Partitioning by Backward Depth Information

The task of coarse partitioning is to partition the state set using backward depth information, and then divide it into many blocks. A block is a subset of state set Q, and is getted by the partition operation accoroding to backward depth information. States in one block are not distinguishable according to the backward depth information after coarse partitioning, so they are instead refined using the hash table presented in the next subsection. There are two steps in coarse partitioning: building backward depth information and partitioning the state set using this information according to proposition 3.

Instead of the maximum level used by Revuz’s algorithm [22], backward depth information is used to initially partition the state set. The maximum level can only be obtained in acyclic automata, which constrains the application range of corresponding minimization algorithms. The time complexity of Revuz’s algorithm is linear because its target is an acyclic automaton, whose underlying graph is a tree. The algorithm in this paper can run in linear time when the automata is acyclic, and can be used in automata with more high topological complexity.

**Fig 3:**
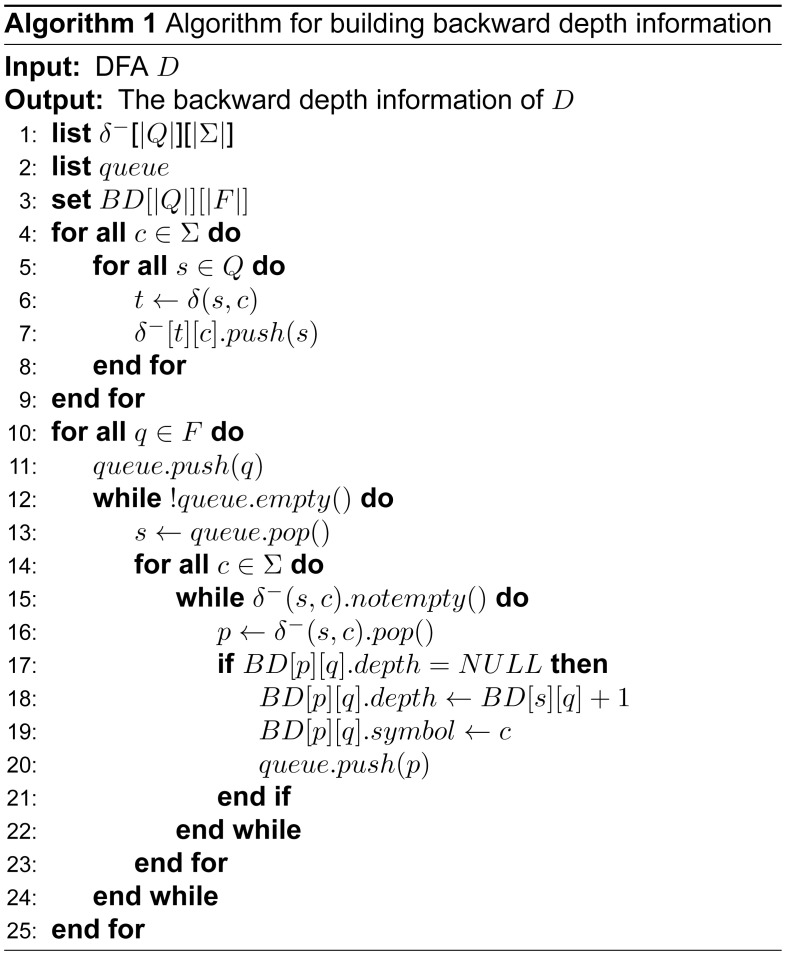
Algorithm for building backward depth information.

The pseudo-code of building backward depth information is shown in Fig 3. When building backward depth information, the reversal DFA is first constructed (lines 4–9). Then, the reversal DFA is traversed in a breadth-first fashion from one certain accepted state *t* using a lexicographic sequence to obtain a coarse partition consisting of blocks. The least number of symbols consumed from state *s* to accepted state *t* is the backward depth of *s* to *t*, denoted as *BD*(*s*, *t*) (lines 10–25). To use backward depth information adequately, the symbols in the shortest path to the state are saved, and then the states that have the same backward depth information are sorted into one block. Thus, the backward depth information is composed by two parts, the backward depth and the last symbol in the shortest path.

**Fig 4:**
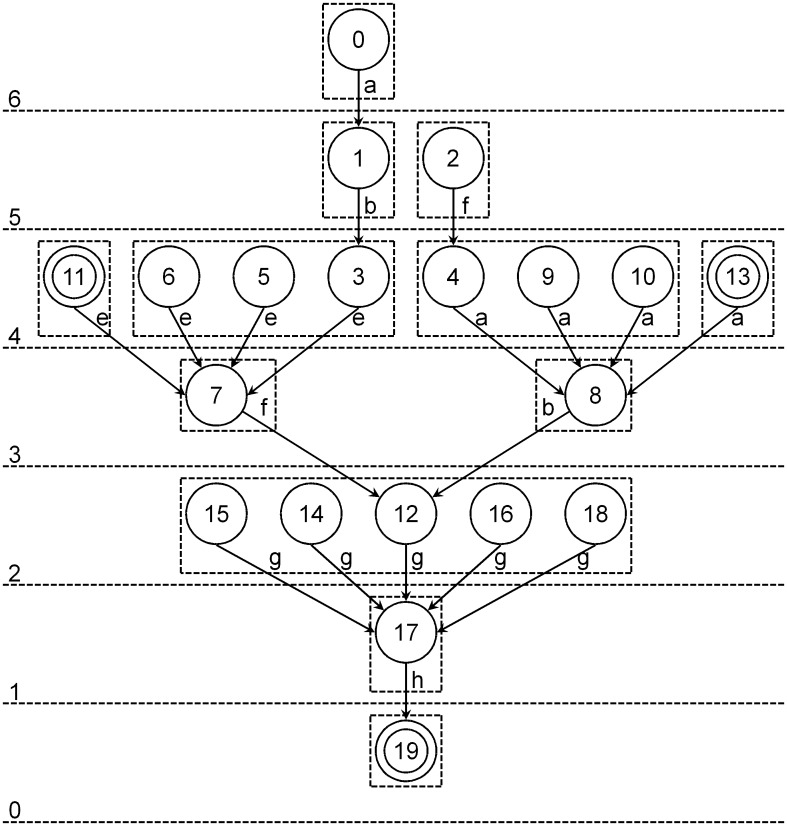
Backward depth information for accepted state “19” in Table 1. The number above the dashed line represents the backward depth, and the states in the same dashed box are in one block. The symbol near the transition is the character consumed in building the shortest path from the state transition graph, and is one part of the backward depth information.

To explain the backward depth information intuitively, the backward depth information for accepted state 19 in the example in Table 1 is shown in Fig 4. The number above the dashed line represents the backward depth to state 19 for the states between the dashed lines, and the states in the same dashed box have the same backward depth information. The backward depth of state 19 is zero, because it consumes no character to reach itself. The states that have a backward depth of four are partitioned into four blocks. This occurs because block generation is determined by the backward depth, the symbol consumed to get to the next state in the graph, and whether the state is an accepted state or not. Block generation based on the backward depth information of an accepted state can be considered to be the shortest path tree whose root is the accepted state obtained from the state transition digraph.

The results of building the backward depth information for the example in Fig 4 are shown in Table 2. After building the backward depth information, we obtain the blocks for different accepted states, and these are shown in the corresponding column for the accepted state. The blocks can be further partitioned according to proposition 3, and the final blocks are shown in the last column in Table 2. Consider states *p* and *q*: if the backward depth information falls within different blocks for any accepted state, they will be arranged into different blocks.

**Table 2:** Backward depth information for automata with the regular expressions (ab.*cd, ef.*gh).

States	Accepted	11		13		18		19		Final block
		BD	Block	BD	Block	BD	Block	BD	Block	
0	0	4,a	0	4,e	0	6,e	0	6,e	0	0
1	0	3,b	1	4,e	0	5,b	1	5,b	1	1
2	0	4,a	0	3,f	1	5,f	2	5,f	2	2
3	0	2,c	2	-1	2	4,e	3	4,e	3	3
4	0	-1	3	2,g	3	4,a	4	4,a	4	4
5	0	2,c	2	-1	2	4,e	3	4,e	3	3
6	0	1,d	4	-1	2	4,e	3	4,e	3	5
7	0	2,c	2	-1	2	3,f	5	3,f	5	6
8	0	-1	3	2,g	3	3,b	6	3,b	6	7
9	0	-1	3	2,g	3	4,a	4	4,a	4	4
10	0	-1	3	1,h	4	4,a	4	4,a	4	8
11	1	0	5	-1	5	4,e	7	4,e	7	9
12	0	-1	3	-1	2	2,c	8	2,g	8	10
13	1	-1	6	-1	6	4,a	9	4,g	9	11
14	0	-1	3	-1	2	2,c	8	2,g	8	10
15	0	-1	3	-1	2	1,d	10	2,g	8	12
16	0	-1	3	-1	2	2,c	8	2,g	8	10
17	0	-1	3	-1	2	2,c	8	1,h	10	13
18	1	-1	7	-1	5	0	11	2,g	11	14
19	1	-1	6	-1	7	2,c	12	0	12	15

**Fig 5:**
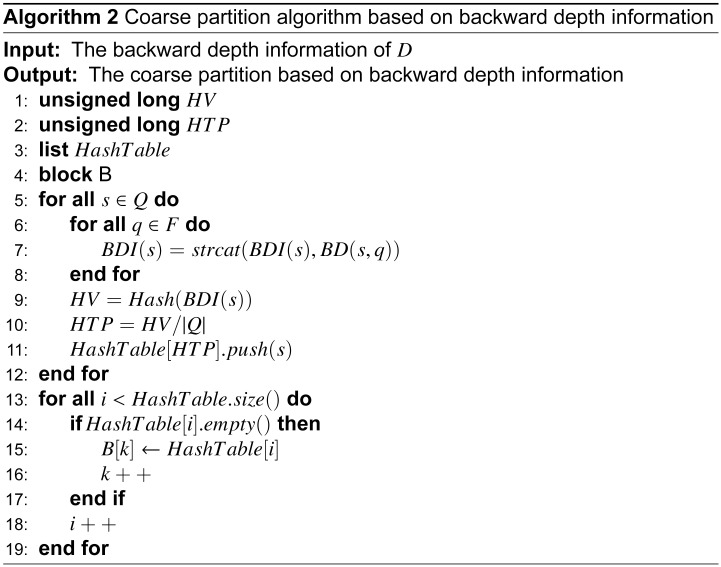
Coarse partition algorithm based on backward depth information.

The coarse partition can be obtained by further block partitioning, and it can be transformed into a comparison of the backward depth information for different states. There are many methods for comparing backward depth information, and the simplest way is direct comparison. However, a direct comparison is costly with respect to time. In this paper, we calculate the hash value (HV) for each state according to the backward information, and then build the hash table. The final blocks can be obtained according to the hash table, that is, if the HVs of two states *p* and *q* are equal, they will be located in the same hash table position (HTP), and then they are assigned to the same block, and vice versa. The pseudo-code of coarse partition algorithm based on backward depth information is shown in Fig 5. The backward depth information of state *s* is denoted as *BDI*(*s*), and it is composed by the connection of the backward depth to different accepted states (line 6–8). The hash values and the their positions in the hash table of backward depth information for each state are computed in line 9–11, and then the coarse partition is generated by the hash table (line 13–19).

In Table 2, blocks are created according to the backward depth information for an accepted state, and the final blocks created according to all backward depth information are listed in the last column. The final block partitioning in the last column may be the final minimization. In other words, the minimal DFA may be found without further computation.

### Refinement Based on Hash Table

When partitioning using backward depth information, not all information about the automata is used. Any two states that belong to different blocks are demonstrably distinguishable, however, it is possible that states in the same block are not equivalent. Therefore, the final minimal DFA can sometimes not be obtained by the first step. To finish the minimization, we use the transition information to refine the blocks in the coarse partition. That is to say, states in one block may be partitioned into new blocks by a hash table of the transition information.

A state in a DFA has many transitions to the next states according to the symbols in the symbol set. A transition is composed of the starting state, symbol, and the ending state, as illustrated in Fig 1. The transitions that have the same starting state are called the state’s transition set. If the transition set of any two states in the same block are different, then the two states are deterministically distinguishable. The refinement of the coarse partition is obtained by a comparison to the transition sets using a hash table. For symbol *a*, if two states *p* and *q* in one block are transferred to different blocks, then *p* and *q* must be distinguishable. The correctness of this approach is proved by proposition 1.

For blocks that have only one state, it is unnecessary to refine them further. Because blocks are coadjacent according to their transitions, many iterations of refinement could be needed for blocks that have many states. Refinement is terminated when no new blocks are generated. If the original DFA is minimal or acyclic, no iterations are needed. Details of the refinement are given in the following.

**Fig 6:**
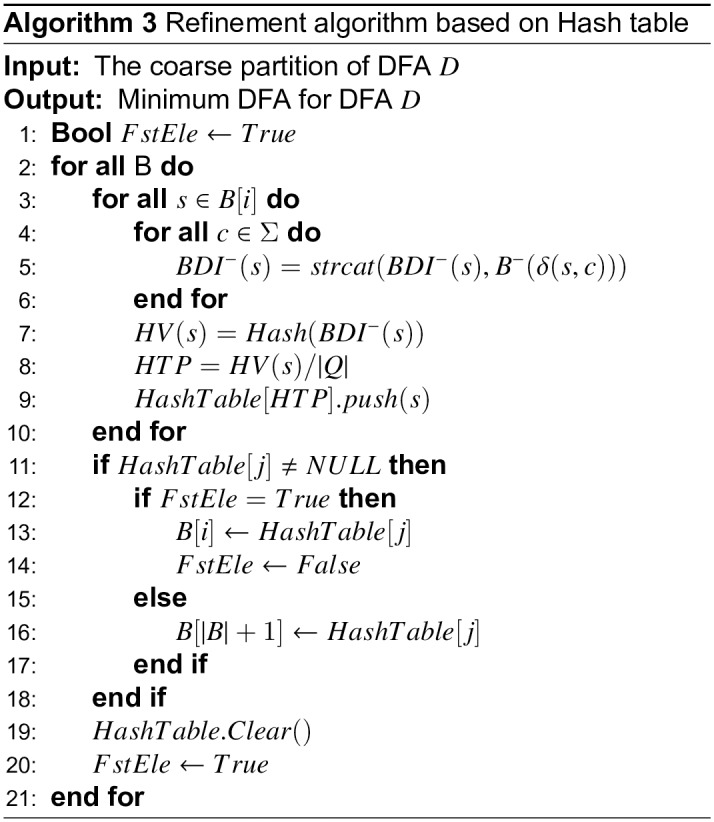
Refinement algorithm based on hash table.

Suppose the cardinality of block *i* is *n*_*i*_. The time complexity of directly comparing any two states in the block is then $O(n_{i}^{2})$. Instead of directly comparing transition sets, we establish a hash table for every block and use it to perform refinements. The pseudo-code for refinement using a hash table is given in Fig 6. The hash value of state *p* is denoted as *HV*(*p*), and the input of the hash calculation is the transition information of the state, which can be expressed as *HV*(*p*) = *Hash*(*BDI*^−^(*p*)) (line 7). The transition information of the state is computed in line 4–6, where *B*^−^(*δ*(*p*, *c*)) represents the information of the block that includes element *δ*(*p*, *c*) (lines 5–9). The position of a state in the corresponding block’s hash table can be obtained by its HV, which is represented as *HTP*(*s*) (line 8). If the positions of the different states are the same, we consider the two states to be equivalent; otherwise, they are distinguishable. If states in a block are distinguishable, the block is divided into multiple blocks (lines 11–20). If a block has only one state, the calculations are ignored. If the number of blocks does not increase during refinement, the algorithm terminates.

Table 3 shows the results of hash table refinement using the example in Table 1. To explain hash table refinement properly, the coarse partition for accepted state 19 instead of the final coarse partition shown in the last subsection is used. In the first refinement, block 3, which includes states 3, 5, and 6, is separated into blocks 3 (states 3 and 5) and 13 (state 6) because the position of state 6 is not the same as that of states 3 and 5 in the hash table. Similarly, state 14 is separated from block 4, and added to a new block 14. State 15 is moved from block 8 to the new block 15. In the second refinement, no new blocks are generated because there are no states in the same block that have different hash table positions, so the refinement is terminated.

**Table 3:** Hash table refinement based on the coarse partition of accepted state 19.

States	19		HV(Fst)	HTP(Fst)	Block(Fst)	HV(Sec)	HTP(Sec)	Final Block
	BD	Block						
0	6,e	0			0			0
1	5,b	1			1			1
2	5,f	2			2			2
3	4,e	3	701180389	7	3	3783612131	5	3
4	4,a	4	2287599808	4	4	233390966	11	4
5	4,e	3	701180389	7	3	3783612131	5	3
6	4,e	3	932010536	2	13			5
7	3,f	5			5			6
8	3,b	6			6			7
9	4,a	4	2287599808	4	4	233390966	11	4
10	4,a	4	285902165	17	14			8
11	4,e	7			7			9
12	2,g	8	248919426	0	8	102449418	15	10
13	4,a	9			9			11
14	2,g	8	248919426	0	8	102449418	15	10
15	2,g	8	59348337	6	15			12
16	2,g	8	248919426	0	8	102449418	15	10
17	1,h	10			10			13
18	2,g	11			11			14
19	0	12			12			15

The advantage of the hash table is that it has lower time complexity: *O*(*n*) compared to the *O*(*n*^2^) of direct comparison. The other noteworthy problem is hash table collision, which occurs when two inputs have the same position although they are different. Distinguishable states will not be found if hash collision occurs among the transition information of these states. In order to guarantee the correctness of the minimal algorithm, an inspection of hash collisions is necessary. This means that the state transition information in the same block must be directly compared once more. The inspections are then performed after the refinements have been terminated, reducing the number of inspections.

To maintain the efficiency of the minimal algorithm, hash collisions should occur with low probability. The frequency of hash collisions is influenced primarily by the hash function and size of the hash table. An exclusive-or hash function [30, 31] is selected for the hash calculations in this paper, and the size of the hash table is the number of states in the DFA. Because the hash table is large enough and an exclusive-or hash function has perfect performance, few collisions occur. Usually, there are no collisions. Even when collisions occur, few inspections are needed because only those states who have the same position in the hash table need to be recalculated.

**Fig 7:**
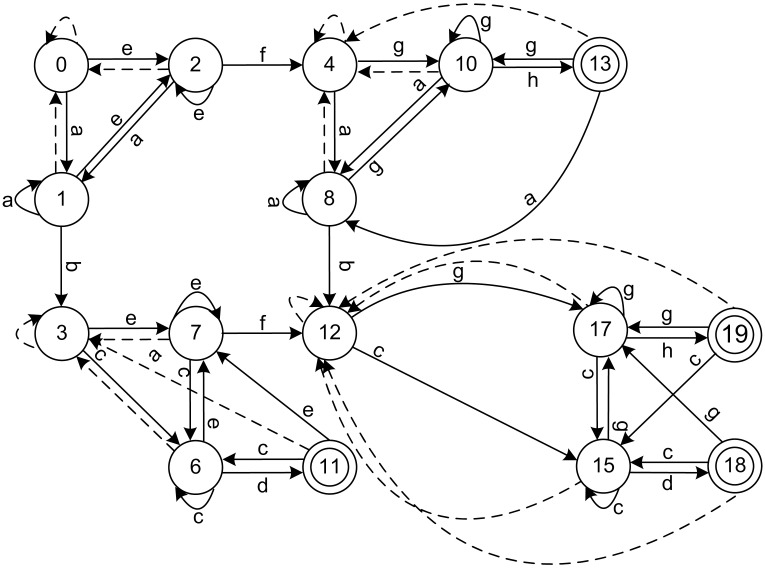
Minimal DFA using the algorithm based on backward depth information for regular expressions (ab.*cd, ef.*gh). The states 4, 9, 12 and 14 are disappeared for the reason that these have been merged into different states, respectively. The minimal DFA is equivalent with original DFA functionally, and is isomorphic with the final block within Table 3.

The final algorithm mainly consists of the backward depth information-based coarse partitioning and hash table refinement. The minimal DFA for the above-mentioned example is shown in Fig 7. As shown in the figure, states 3 and 5 in the original DFA are equivalent and merged into state 3 in the minimal DFA, states 4 and 9 are merged into state 4, and states 12, 14, and 16 are merged into state 12 in the final minimization of the DFA.

## Experiments and Results

To evaluate the performance of the algorithms in this paper, Hopcroft’s algorithm, which known as the most efficient DFA minimization algorithm and has been utilized for various DFAs, was selected for comparison. The proposed algorithm and Hopcroft’s algorithm were implemented in C++ and run under GNU/Linux on a computer with an Intel processor Core i5 (3.2GHz) and 4 GB DDR3 (800 MHz). In order comprehensively and meaningfully compare the two algorithms, three different classes of automata with increasing topological complexity were tested.

At present, there is no standard automata test set for evaluating DFA minimization algorithms. The automata used in this experiment were generated by a workload by Becchi [32], which mainly consists of regular expression generation according to some parameters. An NFA was produced starting from regular expressions using the Thompson method [10], and a DFA was generated by subset construction [12]. Because the topological complexity is mainly determined by wildcard and dot-star terms in regular expression sets, we fix all parameters except for the frequency of wildcard and dot-star terms. The other parameters were set to their default values in the experiment, and the input symbols set Σ was the ASCII alphabet.

**Fig 8:**
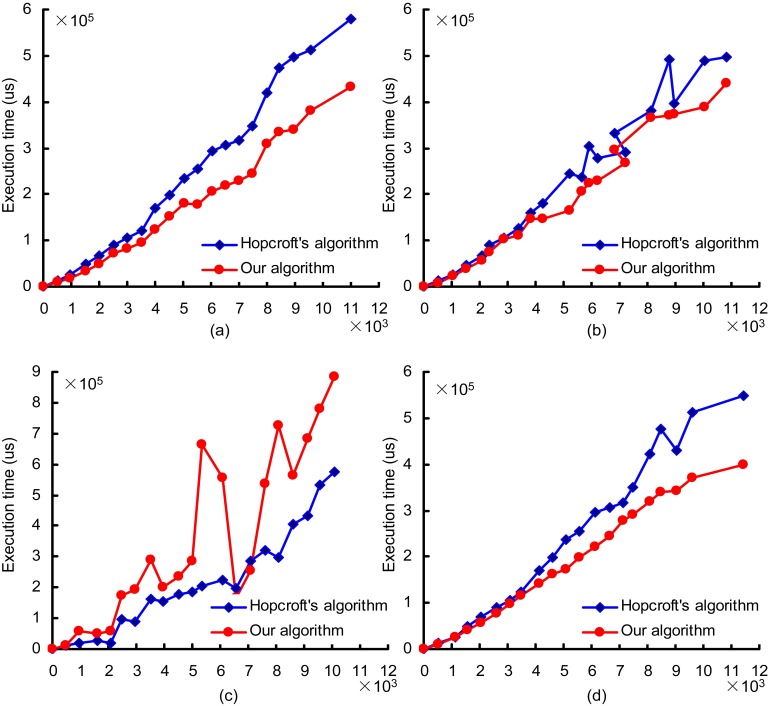
Experimental results for automata with different topological complexities. The horizontal axis represents the number of states in the original DFAs, and the vertical axis indicates the time consumed in microseconds.(a) DFA with an underlying graph consisting of a dictionary, (b) DFA with an underlying graph consisting of a tree, (c) DFA with 1% wildcards and dot-star terms, and (d) DFA with 3% wildcards and dot-star terms.

The experimental results are shown in Fig 8. In this graph, the horizontal axis represents the number of states in the original DFAs, and the vertical axis indicates the time consumed in microseconds. Fig 8(a) and 8(b) show the results for automata with the lowest topological complexity, which do not have any wildcards or dot-star terms in the regular expressions. In this case, the algorithm presented in this paper performs better. This is because the asymptotic time complexity is linear when the automata degrades to an acyclic automata. The results for the automata with medium topological complexity (1% wildcard and dot-star terms) are shown in Fig 8(c). Here, the proposed algorithm does not perform better than Hopcroft’s algorithm. The reason for this is that the proposed algorithm encounters an imperfect partition based on the backward depth information. As shown in Fig 8(d), the proposed algorithm achieves a better time complexity than Hopcroft’s algorithm for the automata with high topological complexity (3% wildcard and dot-star terms).

The time complexity of the proposed algorithm is *O*(*n* + ∑(*n*_*i*_) + *k*), and is composed of three main aspects. First, the time complexity of the coarse partition is *O*(*n*), where *n* represents the number of states in the original DFA. To obtain the backward depth information, the only operation is the construction of the reversal DFA, which consumes *n* periods. The time complexity of the refinements on block *i* without considering hash collisions is *O*(*n*_*i*_), and there may be many blocks that need to be refined by the hash table. Thus, the total time complexity of the hash table refinements without considering hash collisions is *O*(∑(*n*_*i*_)). The hash collision inspections consume fewer cycles because there are not many of them. The time complexity of hash collision inspections is *O*(*k*), where *k* is a random number. The hash table refinements account for the highest proportion of the time complexity because they may be iterated many times. The number of refinement iterations is mainly influenced by the DFA’s topological complexity. If the DFA has an underlying graph with low topological complexity, the states are partitioned primarily by the coarse partition, and the algorithm proposed in this paper will consume comparatively few cycles. For the DFA with high topological complexity, although the state set cannot be partitioned in the coarse partitioning, it is partitioned well in the early stages of the hash table refinements.

## Discussion and Conclusions

The experiments show that the proposed algorithm achieves better time complexity in most cases. This is for three main reasons: the mechanism of coarse partitioning according to backward depth information, the highly efficient comparison of transition information using a hash table, and few hash collisions. The backward depth information that is used to obtain the coarse partition is generated when the reversal DFA is constructed. In the partition refinement to obtain the minimal DFA, the transition information is compared by hash table instead of directly, which decreases the time complexity from $O(n_{i}^{2})$ to *O*(*n*_*i*_).

In addition to the strong quantitative performance results, the proposed method has substantial qualitative advantages, including greater generality and simplicity. Compared to previous minimization algorithms, the backward depth information can be obtained for any type of DFA. Thus, in contrast to previous work, the algorithm proposed in this paper can be used not only on acyclic automata and automata with simple cycles, but also on topologically complicated automata. The maximum level proposed in Revuz’s algorithm is the longest path from the state to the final state, and if the underlying graph has circles, then the maximum level cannot be obtained. All other algorithms except for Hopcroft’s algorithm have similar disadvantages with respect to DFA minimization generality. Furthermore, the algorithm proposed in this paper can be extended to other types of automata; for example, incomplete DFAs. In an incomplete DFA, states may have no transitions for some symbols; however, this has no influence on the implementation of the algorithm. The main operations consist of the building of backward depth information and hash table refinement, and these operate without considering the integrality of the transitions.

In summary, we propose a DFA minimization algorithm based on backward depth information in this paper that is both simple and has better time complexity and greater generality than previous approaches.

## Supporting Information

S1 File The proofs of propositions. S1 File includes Appendixes A and B. Appendix A describes the proof of Proposition 1, and Appendix B gives the proof of Proposition 2.
(PDF)
