# Supplementary material for: Efficient Deterministic Finite Automata Minimization Based on Backward Depth Information
Source: PLoS One. 2016 Nov 2;11(11):e0165864. doi: 10.1371/journal.pone.0165864 (PMC5091862; doi:10.1371/journal.pone.0165864)
Supplement: S1 File — includes Appendixes A and B. Appendix A describes the proof of Proposition 1, and Appendix B gives the proof of Proposition 2. (PDF) [file pone.0165864.s001.pdf]

Appendix A. The Proof of Proposition 1.

Proposition 1. For states $p \in Q$, $q \in Q$, if there exists a symbol a that maintains $\delta(p, a) = s$ and $\delta(q, a) = t$ and furthermore, s and t are distinguishable, then p and q are distinguishable.

Proof. Suppose $\delta(p, a) = s$, $\delta(q, a) = t(s \not\equiv t)$, and $p \equiv q$.

Because $s \not\equiv t$, there must be a word w that satisfies ($\hat{\delta}(s, w) \in F$, $\hat{\delta}(t, w) \notin F$) or ($\hat{\delta}(s, w) \notin F$, $\hat{\delta}(t, w) \in F$).

Therefore, $\hat{\delta}(p, aw) \in F$ and $\hat{\delta}(q, aw) \notin F$, or $\hat{\delta}(p, aw) \notin F$and $\hat{\delta}(q, aw) \in F$.

This means that $p \not\equiv q$, which contradicts the supposition. Hence, proposition 1 is proved. □

Appendix B. The Proof of Proposition 2.

Proposition 2. If the backward depths of two states p and q for any accepted state t are different, p and q must be distinguishable. Formally, if $BD(p, t) \neq BD(q, t)$, then $p \not\equiv q$.

Proof. Because $BD(p, t) \neq BD(q, t)$, there exist words w_i and w_j that maintain $\hat{\delta}(p, w_i) = t$ and $\hat{\delta}(q, w_j) = t$, respectively, where $|w_i| \neq |w_j|$.

If $|w_i| < |w_j|$, then $\hat{\delta}(p, w_i) = t$ and $\hat{\delta}(q, w_j) \neq t$.

Thus, p and q are distinguishable ($p \not\equiv q$). □
